# Chronic Pre‐Trauma Corticosterone Attenuates Trauma Memory Formation and Alters Gene Expression in Glucocorticoid Signalling Pathways

**DOI:** 10.1111/gbb.70067

**Published:** 2026-09-07

**Authors:** Gia Kutelia, Zurab Kuchukashvili, Rusiko Ansiani, Roza Bukia, Manana Chikovani, Butsiko Chkhartishvili, Nanuli Doreulee

**Affiliations:** ^1^ Department of Biology, Faculty of Exact and Natural Sciences Ivane Javakhishvili Tbilisi State University Tbilisi Georgia; ^2^ Department of Biocybernetic Systems Vladimer Chavchanidze Institute of Cybernetics of the Georgian Technical University Tbilisi Georgia

**Keywords:** animal models, corticosteroid receptors, glucocorticoids, memory consolidation, PTSD, trauma memory

## Abstract

Glucocorticoid signalling plays a central role in the processing of traumatic memories and contributes to adaptive and pathological responses to stress, with dysregulation frequently reported in trauma‐related conditions such as post‐traumatic stress disorder (PTSD). A key stage in trauma memory formation is the early consolidation window, during which aversive experiences are stabilised into long‐term memory through protein synthesis and activity‐dependent gene expression. To investigate how glucocorticoid levels during trauma exposure shape this process, we examined the behavioural and molecular consequences of acute and chronic corticosterone elevation, the rodent analogue of cortisol. Adult male mice received either repeated corticosterone injections for 5 days prior to fear conditioning (FC) or vehicle injections for 5 days followed by a single corticosterone injection immediately before FC. Two hours after FC, we quantified expression of genes encoding mineralocorticoid receptors (*Nr3c2*, MR), glucocorticoid receptors (*Nr3c1*, GR), and FK506‐binding protein 5 (*Fkbp5*) in the amygdala, hippocampus, and anterior cingulate cortex (ACC). Trauma memory was assessed 24 h later by measuring freezing and exploratory behaviour to conditioned cues in separate experimental cohorts. Chronic corticosterone exposure was associated with attenuated conditioning‐related behavioural responses, whereas acute corticosterone did not alter these responses. These effects were accompanied by region‐specific alterations in glucocorticoid‐signalling gene expression, most notably coordinated regulation of hippocampal and ACC *Nr3c2* following FC. We propose that this regional *Nr3c2* transcriptional signature represents an early molecular adaptation to prolonged glucocorticoid exposure that may alter hippocampal–prefrontal communication during trauma‐memory formation.

## Graphical abstract

The graphical abstract outlines the experimental workflow and key findings, showing how chronic or acute corticosterone elevations alter FC‐induced molecular and behavioural changes during trauma memory formation, consolidation, and recall. Abbreviations: Cort.—Corticosterone; FC—Fear Conditioning; PRPs—Plasticity‐Related Proteins.

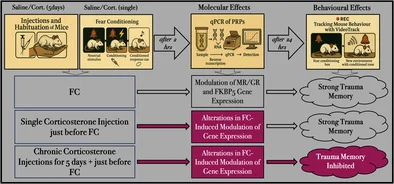

## 1 Introduction

Glucocorticoids are central regulators of the stress response, and their dysregulation has been implicated in numerous stress‐related disorders [1, 2, 3]. Beyond their well‐known metabolic actions, glucocorticoids facilitate adaptive physiological responses by mobilising energy resources during stress [4, 5] and suppressing immune activity to limit inflammation and prevent excessive physiological strain [3]. Extreme or overwhelming stress, commonly defined as trauma, is often associated with the formation of persistent and intrusive traumatic memories [6]. Yet the precise role of glucocorticoids in trauma‐memory formation remains unresolved, as existing findings are frequently inconsistent [7, 8, 9, 10, 11]. Individuals with PTSD typically show altered cortisol dynamics and glucocorticoid sensitivity relative to healthy controls, particularly during and following trauma‐memory retrieval. Some studies report elevated cortisol levels and disrupted GR sensitivity during recall [12, 13, 14, 15], whereas others describe reduced basal cortisol and diminished stress responsivity [16, 17]. These divergent results highlight substantial heterogeneity in glucocorticoid signalling in PTSD [18], indicating that cortisol alterations are likely context‐ and state‐dependent rather than uniform across individuals. Whether such changes reflect adaptive compensatory processes or instead contribute directly to PTSD pathophysiology remains unclear [19]. Importantly, fluctuations in cortisol levels during trauma‐memory retrieval may alter the balance between MR and GR activation, thereby affecting memory reactivation, reconsolidation, and the long‐term persistence of traumatic memories.

At the neural level, glucocorticoid effects are mediated by the coordinated activation of MRs and GRs, whose relative engagement determines whether a *plasticity window* is opened or dysregulated [1, 20, 21, 22]. Due to their high affinity for cortisol, MRs are predominantly occupied under basal and moderate stress conditions and act as primary responders to rising glucocorticoid levels [23, 24]. MR activation facilitates emotional regulation, enhances glutamatergic transmission, lowers encoding thresholds, and promotes synaptic plasticity, thereby opening the plasticity gate and supporting efficient memory formation and consolidation when stress is contextually relevant [25, 26]. In this framework, MRs define the upper boundary of adaptive stress by setting receptor‐binding thresholds that distinguish normal from overwhelming stress exposure [27]. In contrast, GRs are primarily recruited at higher corticosterone concentrations and are minimally engaged under baseline conditions [28, 29, 30, 31]. GR activation shifts neural processing towards a stress‐reactive state, suppresses or alters ongoing encoding, and initiates negative feedback on the hypothalamic–pituitary–adrenal (HPA) axis [5, 24, 25, 32]. Excessive or prolonged GR activation may therefore interfere with normal trauma memory shaping by disrupting the temporal closure of the plasticity window and altering transcriptional pathways required for stable consolidation. Maintaining an appropriate MR/GR balance is thus critical for adaptive stress responses and healthy trauma memory processing [33]. FK506‐binding protein 5 (FKBP5) is a key intracellular regulator of GR signalling, buffering excessive glucocorticoid receptor activation and shaping cellular responses to elevated cortisol levels. Under conditions of increased glucocorticoid exposure, *Fkbp5* expression is induced and attenuates glucocorticoid signalling by impairing the nuclear translocation of the cortisol–GR complex, thereby limiting downstream transcriptional activity [34, 35, 36]. Consistent with this role, Li et al. [37] demonstrated that the GR–FKBP51 complex is significantly elevated in both PTSD patients and fear‐conditioned mice, where it is associated with impaired GR signalling, reduced GR nuclear translocation, and enhanced fear‐related behaviours. Importantly, disruption of GR–FKBP51 binding reduced freezing behaviour and reversed stress‐associated molecular alterations. Within the MR–GR plasticity framework, FKBP5 may therefore function as a molecular brake that constrains prolonged GR‐driven plasticity and protects against excessive or maladaptive trauma‐related memory formation.

In this study, we used real‐time quantitative polymerase chain reaction (qPCR) to examine how acute and chronic administration of a high dose of corticosterone (20 mg/kg; [38]) affects the expression of genes encoding the MR (*Nr3c2*), GR (*Nr3c1*), and FKBP5 (*Fkbp5*) 2 h after FC as this period corresponds to the early phase of memory consolidation, characterized by active transcriptional and plasticity‐related processes involved in long‐term memory formation [39, 40, 41]. Specifically, we aimed to determine how experimentally elevating corticosterone levels for five consecutive days before trauma (chronic corticosterone) or acutely at the time of trauma (acute corticosterone) alters the relative expression of these key components of glucocorticoid signalling. This approach provides insight into how different temporal patterns of corticosterone exposure modulate receptor‐ and co‐chaperone‐mediated signalling, and how these transcriptional responses differ following acute versus repeated corticosterone exposure in the presence or absence of FC. We focused on three brain regions that play critical roles in trauma‐related memory processing: the hippocampus, amygdala, and ACC [42]. To assess whether these molecular alterations translate into functional consequences, we further examined the effects of corticosterone on trauma‐memory formation by evaluating fear memory retrieval 24 h after FC in behavioural experiments.

## 2 Materials and Methods

### 2.1 Animals

Young adult male non‐linear (outbred) white laboratory wild‐type mice aged 4–6 months and weighing approximately 30 g were used in all experiments. The mice were purchased from the Aleksandre Natishvili Institute of Morphology, Ivane Javakhishvili Tbilisi State University (78 Beliashvili Street, Tbilisi, Georgia; https://morphology.tsu.ge/). They originated from Swiss Webster stock and were bred and maintained as an institutional outbred colony using breeding practices that minimised inbreeding. Following purchase, the animals were allowed to acclimatise for 1 week and were then randomly assigned to the experimental groups. During the final five consecutive days before behavioural testing, mice were habituated to handling, the experimenter, and the experimental environment to minimise handling‐ and novelty‐related stress. Male mice were selected to minimise potential variability associated with the female estrous cycle and its interactions with corticosterone‐mediated effects. All experimental procedures were approved by a board of professors from the Department of Biology, Faculty of Exact and Natural Sciences, Ivane Javakhishvili Tbilisi State University (FR/22‐26152), and were performed in accordance with the Law of Georgia on Health Care, Directive 2010/63/EU of the European Parliament, and the European Convention for the Protection of Vertebrate Animals Used for Experimental and Other Scientific Purposes. Animals were housed in a temperature‐controlled vivarium (23°C ± 1°C, 50%–60% humidity) under a 12 h light/dark cycle, with food and water available ad libitum. Efforts were made to minimise the number of animals and their suffering.

### 2.2 Experimental Groups

Animals were randomly assigned to one of six experimental groups to assess the effects of corticosterone administration on FC‐associated behavioural and molecular responses. To control for handling, injection frequency, and vehicle exposure, all mice received one subcutaneous injection daily for five consecutive days before the experimental session and one additional injection immediately before FC or chamber exposure. The groups were as follows:

1. Control (No CORT, No FC): Mice received vehicle (undiluted DMSO; Sigma‐Aldrich, D8418, purity ≥ 99.9%) during the 5‐day pretreatment period and an additional vehicle injection immediately before placement in the conditioning chamber. No footshocks were administered. This group served as the baseline control for molecular measurements.
2. FC (No CORT + FC): Mice received vehicle during the 5‐day pretreatment period and an additional vehicle injection immediately before FC, followed by five tone–footshock pairings as described below. This group served as the primary comparison for trauma‐induced behavioural and molecular changes.
3. Acute CORT + FC: Mice received vehicle during the 5‐day pretreatment period, followed by a single corticosterone injection immediately before FC. They were then subjected to the standard tone–footshock protocol. This group was used to investigate the effect of acute corticosterone administration on trauma‐memory formation.
4. Chronic CORT + FC: Mice received corticosterone once daily for five consecutive days before FC, followed by an additional corticosterone injection immediately before FC. They were then subjected to the standard tone–footshock protocol. This group modelled the effects of repeated glucocorticoid exposure before trauma.
5. Acute CORT (No FC): Mice received vehicle during the 5‐day pretreatment period, followed by a single corticosterone injection immediately before placement in the conditioning chamber. No footshocks were administered. This group allowed assessment of acute corticosterone effects in the absence of trauma.
6. Chronic CORT (No FC): Mice received corticosterone once daily for five consecutive days before chamber exposure, followed by an additional corticosterone injection immediately before placement in the conditioning chamber. No footshocks were administered. This group was included to assess the effects of repeated corticosterone exposure in the absence of trauma.

### 2.3 Fear Conditioning

Preliminary experiments comparing tone‐cued and contextual FC were performed to optimise the protocol, and the paradigm producing stronger trauma‐associated memory responses was selected for the corticosterone studies. Mice were subjected to auditory cued (tone) FC in standard conditioning chambers. Prior to FC, animals underwent 5 days of habituation to the experimenter and the experimental chambers. On the day of FC, mice were acclimated to the experimental room for at least 30 min before testing. Each conditioning session lasted 7 min. Mice were placed individually in a conditioning chamber and allowed to freely explore for a 120‐s habituation period. Shock reactivity was monitored qualitatively during training by assessing vocalisation responses to footshocks. At the selected shock intensity, comparable vocalisation patterns were observed across groups, indicating a similar perception of the unconditioned stimulus. The FC apparatus used in this study was custom‐built in our laboratory based on the guidelines described by Jan Bureš, Olga Burešová, and Joseph P. Huston in Techniques and Basic Experiments for the Study of Brain and Behavior Bureš [43]. The conditioning chamber measured 15 × 20 cm and was equipped with a stainless‐steel shock grid capable of delivering AC footshocks ranging from 0.1 to 4.0 mA in 0.1 mA increments, with shock onset and termination controlled manually or via software. The apparatus was housed within a sound‐attenuating chamber and equipped with a camera for behavioural recording. Behavioural tracking and analysis were performed using VideoTrack rodent behaviour tracking software (Viewpoint, France). FC consisted of five tone‐footshock pairings. Each auditory cue was a 30‐s frequency‐modulated ascending sweep (5–15 kHz), co‐terminating with a 2‐s footshock (0.8 mA, constant current) delivered through the grid floor. After the final pairing, mice remained in the chamber for an additional 60 s before being returned to their home cages.

### 2.4 Injections

Corticosterone (EMD Millipore Corp., USA) was dissolved in undiluted DMSO (Sigma‐Aldrich, D8418, purity ≥ 99.9%) without additional solvent or dilution and administered subcutaneously at a dose of 20 mg/kg (equivalent to 0.6 mg for a 30 g mouse). Accordingly, each 30 g animal received 50 μL of corticosterone solution or vehicle (undiluted DMSO; Sigma‐Aldrich, D8418, purity ≥ 99.9%) per injection, according to the assigned experimental group. All animals received the same number of subcutaneous injections according to the experimental design to ensure equivalent handling, injection frequency, and vehicle exposure across groups. To control for endogenous corticosterone fluctuations, all injections and behavioural procedures were performed during the light phase (ZT4–ZT6), when basal corticosterone levels are minimal.

### 2.5 Recording and Quantification of Behaviour

Behavioural responses were recorded and analysed using the VideoTrack system (ViewPoint, France). Freezing behaviour, defined as the absence of all movement except respiration, was the primary measure of fear memory, while locomotor activity (distance travelled and movement duration) was recorded to assess exploration and motor function. Conditioning and testing chambers were illuminated under standard lighting to ensure proper video tracking and animal visibility. To establish baseline tone reactivity, mice were exposed to the auditory cue 24 h before FC in a novel chamber differing in size (30 × 40 cm) and wall/floor texture. During this baseline session, mice received five 30‐s ascending tones (5–15 kHz) without footshock, and freezing and activity were quantified using VideoTrack software. Memory retrieval was tested 24 h after FC in the same context as the pre‐conditioning tone exposure, again without footshocks, using the identical tone protocol. Responses to tone—total freezing duration and locomotor activity across the session were quantified semi‐automatically by VideoTrack based on predefined inactivity thresholds. This two‐context design enabled assessment of cue‐specific memory retrieval independent of contextual fear, ensuring that observed behavioural changes reflected learned associations with the auditory stimulus.

### 2.6 Tissue Collection and qPCR Analysis

Two hours after FC, animals were deeply anesthetised with Calypsol (ketamine, Gedeon Richter) and Xylazine (2%, Bioveta a.s.) at a 9:1 ratio, followed by perfusion with phosphate‐buffered saline (PBS; EMD Millipore Corp., USA). Brains were dissected, transferred to ice‐cold PBS, sliced into 400 μm‐thick coronal sections using a vibroslicer (Campden Instruments), *and the hippocampus, amygdala, and* ACC *were isolated* by microdissection under a stereomicroscope using a sterile scalpel on an ice‐cold surface, according to the Paxinos and Franklin Mouse Brain Atlas (5th Edition, 2019). The approximate anteroposterior coordinates relative to bregma were +1.98 to −0.10 mm for the ACC, −0.70 to −3.80 mm for the amygdala, and −1.06 to −3.88 mm for the hippocampus. Total RNA was extracted from each brain region using the GeneJET RNA Purification Kit (Thermo Fisher Scientific) following the manufacturer's instructions. Tissue samples were homogenised in lysis buffer, and RNA was eluted in 30–50 μL of RNase‐free water. RNA quantity and purity were assessed spectrophotometrically using a NanoDrop device, with A260/280 ratios close to 2.0 considered acceptable, and verified using a Qubit 4 Fluorometer. RNA integrity was further confirmed by electrophoresis on a 1% agarose gel containing ethidium bromide; only samples displaying sharp 28S and 18S rRNA bands without degradation were used for downstream analysis. Complementary DNA (cDNA) was synthesized from total RNA using the Thermo Scientific Maxima H Minus cDNA Synthesis Master Mix with dsDNase (Thermo Fisher Scientific), which contains a combination of oligo(dT)18 and random hexamer primers, in a final volume of 20 μL. Reverse transcription was performed at 25°C for 10 min, 50°C for 10 min, and 85°C for 5 min. cDNA was stored at −80°C until use. Quantitative real‐time PCR (qPCR) was performed using gene‐specific primers for mineralocorticoid receptor (MR; Nr3c2, Assay ID Mm01241596_m1), glucocorticoid receptor (GR; Nr3c1, Assay ID Mm00433832_m1), and FKBP5 (*Fkbp5*, Assay ID Mm00487406_m1), with hypoxanthine‐guanine phosphoribosyltransferase (HPRT; *Hprt*, Assay ID Mm00446968_m1) as the housekeeping gene. Predesigned TaqMan Gene Expression Assays were obtained from Thermo Fisher Scientific. Each 20‐μL qPCR reaction contained 10 μL of TaqMan Fast Advanced Master Mix, 1 μL of the corresponding 20× TaqMan Gene Expression Assay, 2 μL of 1:10 diluted cDNA, and 7 μL of nuclease‐free water. Amplifications were performed in triplicate on a QuantStudio 5 Real‐Time PCR System (Applied Biosystems) with the following cycling conditions: 50°C for 2 min, 95°C for 2 min, followed by 40 cycles of 95°C for 1 s and 60°C for 20 s. Relative expression was calculated using the 2^−ΔΔCt^ method, with the mean of the No CORT, No FC group used as the calibrator for each gene and brain region. Fold‐change values were log‐transformed before statistical analysis and graphical presentation. Non‐template controls were included to detect contamination, and primer efficiencies were verified to be within 90–110% before analysis.

### 2.7 Statistical Analysis

Behavioural data were analysed in GraphPad Prism using a mixed‐effects model (REML) with time (before FC vs. after FC) as a within‐subject repeated‐measures factor and treatment group as a between‐subject factor. This approach was chosen to account for repeated measurements within animals and unequal sample sizes across groups. Following the mixed‐effects analysis, Tukey's multiple comparisons test was applied to compare pre‐ versus post‐FC behaviour within each group. Because altered behavioural responses were observed specifically following chronic CORT treatment combined with FC, the Chronic CORT (No FC) group was additionally examined to determine whether chronic CORT alone altered freezing or exploratory activity in the absence of FC. Results are reported as least‐squares (LS) means with corresponding mean differences, 95% confidence intervals, and adjusted *p*‐values. Statistical significance was set at *α* = 0.05.

Gene expression data were analysed separately for each brain region using an ordinary two‐way analysis of variance (ANOVA), with Treatment (No CORT, Acute CORT, Chronic CORT) and Experience (No FC, FC) as fixed factors in GraphPad Prism. The analysis evaluated the main effects of Treatment and Experience and the Treatment × Experience interaction. Tukey's multiple‐comparisons test was subsequently performed for all analyses to compare treatment groups within each Experience condition and Experience conditions within each Treatment group. These pairwise comparisons were performed irrespective of the statistical significance of the corresponding omnibus main effects or interaction, with multiplicity‐adjusted *p* values reported for all comparisons. Results are reported as least‐squares (LS) means with corresponding mean differences, 95% confidence intervals, and adjusted *p* values. Statistical significance was set at α = 0.05. All analyses were conducted using GraphPad Prism.

## 3 Results

### 3.1 Behavioural Effects of Acute and Chronic Corticosterone on Fear Conditioning

**FIGURE 1:**
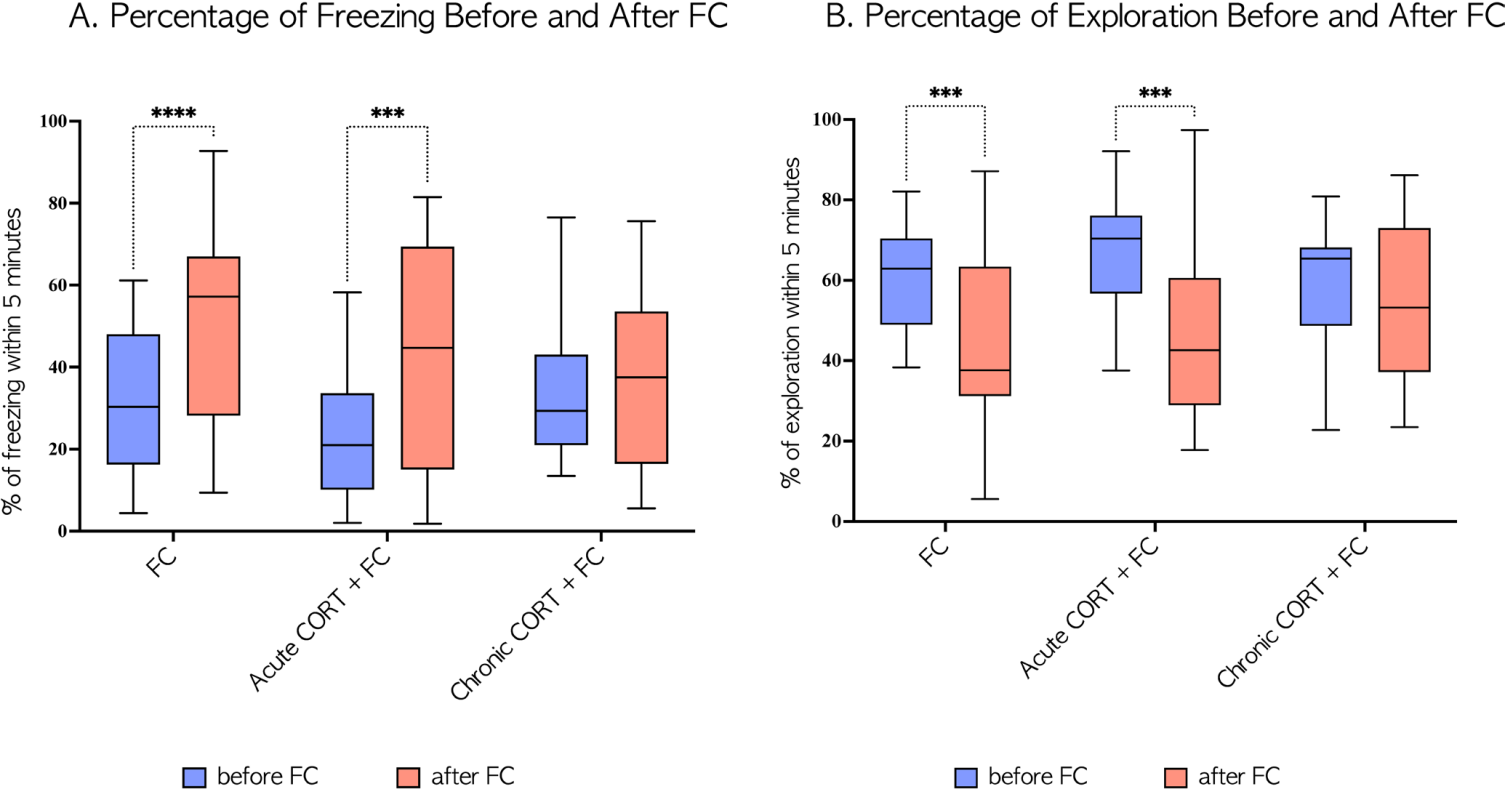
(A, B) Fear‐related behavioural responses before and after FC. (A) Percentage of freezing and (B) percentage of exploratory activity measured before and 24 h after FC in FC, Acute CORT + FC, and Chronic CORT + FC groups. FC and acute corticosterone‐treated mice showed significantly increased freezing and reduced exploration after FC, whereas chronic corticosterone exposure attenuated these behavioural effects. Data are presented as box‐and‐whisker plots showing the median, interquartile range, and minimum‐to‐maximum values. Statistical analysis was performed using a mixed‐effects model fitted by restricted maximum likelihood followed by Tukey's multiple comparisons test. ***p < 0.001, ****p < 0.0001.

FC induced robust behavioural alterations in freezing and exploratory activity, whereas chronic corticosterone exposure attenuated these effects (Figure 1A,B). Behavioural data were analysed using a mixed‐effects model fitted by restricted maximum likelihood, with Time as a repeated‐measures factor and Treatment group as a between‐subject factor. For freezing behaviour, the analysis revealed a significant main effect of Time [*F*(1,60) = 33.36, *p* < 0.0001] and a significant Treatment × Time interaction [*F*(2,60) = 3.716, *p* = 0.0301]. In contrast, the main effect of Treatment was not significant [*F*(2,61) = 0.8839, *p* = 0.4184]. Tukey's multiple‐comparisons test demonstrated significant increases in freezing after FC in both the FC group (mean difference = 15.95, 95% CI [8.246, 23.65], adjusted *p* < 0.001; *n* = 24/group) and the Acute CORT + FC group (mean difference = 21.23, 95% CI [12.15, 30.31], adjusted *p* < 0.001; *n* = 18 before FC, *n* = 17 after FC), whereas the Chronic CORT + FC group did not show a significant increase in freezing following FC (mean difference = 5.974, 95% CI [−2.073, 14.02], adjusted *p* = 0.143; *n* = 22/group). No significant differences between experimental groups were observed either before or after FC (all adjusted *p* > 0.05). Additionally, in the Chronic CORT (No FC) group, freezing behaviour did not significantly differ between animals tested before and after chamber exposure without footshocks (*n* = 15/group; estimate = −9.02 ± 7.45, z = −1.21, *p* = 0.226). Consistently, post hoc Tukey's multiple‐comparisons test showed no significant difference between the Before FC and After FC conditions (*p* = 0.236).

Similarly, for exploratory activity, the mixed‐effects analysis revealed a significant main effect of Time [*F*(1,60) = 36.06, *p* < 0.001] and a significant Treatment × Time interaction [*F*(2,60) = 3.385, *p* = 0.0400], whereas the main effect of Treatment was not significant [*F*(2,61) = 0.6169, *p* = 0.5430]. Tukey's multiple‐comparisons test showed that exploratory activity significantly decreased after FC in both the FC group (mean difference = −19.33, 95% CI [−27.62, −11.04], adjusted *p* < 0.0001; *n* = 24/group) and the Acute CORT + FC group (mean difference = −20.24, 95% CI [−30.03, −10.46], adjusted *p* = 0.0001; *n* = 18 before FC, *n* = 17 after FC). In contrast, the Chronic CORT + FC group showed no significant reduction in exploration following FC (mean difference = −5.115, 95% CI [−13.77, 3.544], adjusted *p* = 0.2420; *n* = 22/group). No significant between‐group differences were detected either before or after FC (all adjusted *p* > 0.05). Likewise, the Chronic CORT (No FC) showed no significant change in exploratory activity before versus after chamber exposure without footshocks (*n* = 15/group; z = 1.05, *p* = 0.294; Tukey's post hoc *p* = 0.301).

### 3.2 Expression of Glucocorticoid‐Signalling Genes

To determine whether corticosterone treatment and FC experience independently or interactively influenced the expression of *Nr3c2*, *Nr3c1*, and *Fkbp5* in the hippocampus, amygdala, and ACC, qPCR data were analysed using an ordinary two‐way ANOVA with Treatment (No CORT, Acute CORT, and Chronic CORT) and Experience (No FC and FC) as fixed factors. Tukey's multiple‐comparisons test was subsequently performed for all analyses to compare treatment groups within each Experience condition and No FC versus FC within each Treatment group, with multiplicity‐adjusted *p* values reported for these pairwise comparisons.

#### 3.2.1 Relative Expression of *Nr3c2*

**FIGURE 2:**
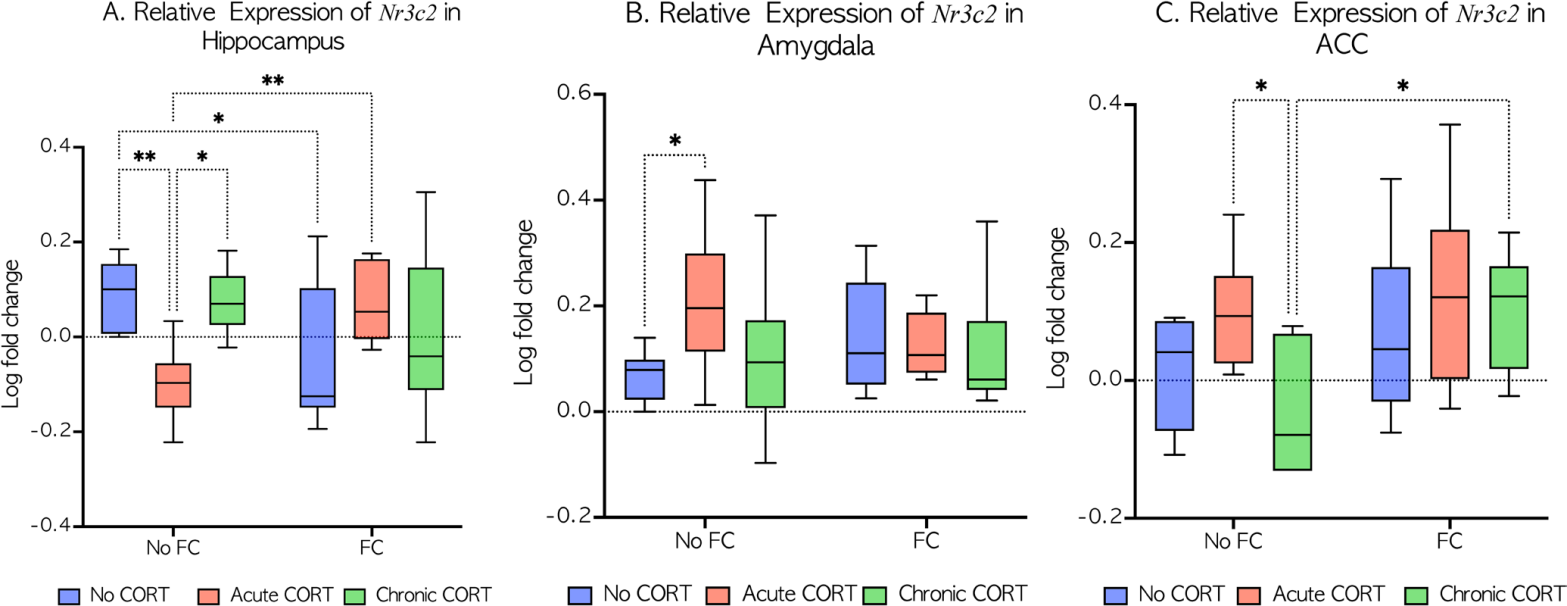
Effects of corticosterone treatment and FC experience on Nr3c2 mRNA expression in the hippocampus, amygdala, and ACC. RT‐qPCR quantified relative Nr3c2 mRNA expression in the hippocampus (A), amygdala (B), and ACC (C) of mice receiving vehicle (No CORT), acute corticosterone (Acute CORT), or chronic corticosterone (Chronic CORT) treatment in the absence (No FC) or presence (FC) of FC. Gene expression was normalised to Hprt. Data are presented as box‐and‐whisker plots showing the median, interquartile range, and minimum‐to‐maximum values. Statistical analyses were performed separately for each brain region using ordinary two‐way ANOVA with Treatment (No CORT, Acute CORT, Chronic CORT) and Experience (No FC, FC) as fixed factors, followed by Tukey's multiple‐comparisons test. *n* = 9/group, except Chronic CORT (No FC) (*n* = 6) and Chronic CORT + FC for ACC (*n* = 8 due to tissue damage). Significant pairwise comparisons are indicated by brackets (*p < 0.05, **p < 0.01).

In the hippocampus, the analysis revealed a significant Treatment × Experience interaction (*F*(2,45) = 9.045, *p* = 0.0005), whereas neither the main effect of Treatment (*F*(2,45) = 1.018, *p* = 0.3694) nor Experience (*F*(1,45) = 0.1039, *p* = 0.7487) was statistically significant (Figure 2A). These findings indicate that the effect of corticosterone treatment on hippocampal *Nr3c2* expression differed according to FC experience. To further characterize this interaction, Tukey's multiple‐comparisons test was performed. Within the No FC condition, acute corticosterone treatment significantly reduced *Nr3c2* expression compared with vehicle‐treated mice (mean difference = 0.1872, 95% CI = 0.06035‐0.3141, adjusted *p* = 0.0024). Likewise, *Nr3c2* expression was significantly lower in the acute corticosterone group than in the chronic corticosterone group (mean difference = −0.1752, 95% CI = −0.3171 to −0.03331, adjusted *p* = 0.0122). No significant differences among treatment groups were observed following FC. Comparison of Experience levels demonstrated significantly lower *Nr3c2* expression in vehicle‐treated mice following FC than under basal conditions (mean difference = 0.1277, 95% CI = 0.02222–0.2331, adjusted *p* = 0.0188). In contrast, acute corticosterone‐treated mice exhibited significantly higher *Nr3c2* expression after FC than in the corresponding No FC group (mean difference = −0.1705, 95% CI = −0.2760 to −0.06509, adjusted *p* = 0.0021). No significant effect of Experience was detected within the chronic corticosterone group.

In the amygdala, two‐way ANOVA revealed no significant Treatment × Experience interaction (*F*(2,45) = 2.837, *p* = 0.0691), and neither the main effect of Treatment (*F*(2,45) = 1.749, *p* = 0.1856) nor Experience (*F*(1,45) = 0.04964, *p* = 0.8247) reached statistical significance (Figure 2B). Overall, these findings indicate that *Nr3c2* expression in the amygdala was not substantially altered by corticosterone treatment or FC experience. Although the overall ANOVA did not identify significant main effects or an interaction, Tukey's multiple‐comparisons test detected a significant difference within the No FC condition, where acute corticosterone‐treated mice exhibited higher *Nr3c2* expression than vehicle‐treated animals (mean difference = −0.1377, 95% CI = −0.2547 to −0.02070, adjusted *p* = 0.0176). No additional pairwise comparisons reached statistical significance.

Analysis of *Nr3c2* expression in the ACC demonstrated significant main effects of both Experience (*F*(1,44) = 7.067, *p* = 0.0109) and Treatment (*F*(2,44) = 3.392, *p* = 0.0427), whereas the Treatment × Experience interaction was not significant (*F*(2,44) = 1.452, *p* = 0.2451) (Figure 2C). These findings indicate that corticosterone treatment and FC experience independently influenced ACC *Nr3c2* expression. Post hoc Tukey's multiple‐comparisons analysis demonstrated that, within the No FC condition, *Nr3c2* expression differed significantly between acute and chronic corticosterone‐treated mice (mean difference = 0.1449, 95% CI = 0.01575–0.2740, adjusted *p* = 0.0247). Furthermore, within the chronic corticosterone treatment group, fear‐conditioned mice exhibited significantly higher *Nr3c2* expression than non‐fear‐conditioned mice (mean difference = −0.1469, 95% CI = −0.2568 to −0.03690, adjusted *p* = 0.0100). No additional pairwise comparisons reached statistical significance.

Collectively, these findings demonstrate distinct patterns of *Nr3c2* regulation across the three brain regions examined. The hippocampus exhibited a significant interaction between corticosterone treatment and FC experience, indicating that the transcriptional response of *Nr3c2* to corticosterone depended on prior behavioural experience. Specifically, acute corticosterone significantly reduced hippocampal *Nr3c2* expression under basal conditions, whereas this effect was not observed following FC, which was associated with opposite changes in *Nr3c2* expression in vehicle‐ and acute corticosterone‐treated animals. In contrast, amygdalar *Nr3c2* expression remained comparatively stable across experimental conditions, with only a single significant pairwise difference detected in non‐fear‐conditioned animals and no significant overall effects of Treatment or Experience. The ACC displayed a distinct response pattern characterized by independent effects of corticosterone treatment and FC experience, with differences between acute and chronic corticosterone treatment under basal conditions and increased *Nr3c2* expression following FC in chronically treated mice. Overall, these results indicate that glucocorticoid regulation of *Nr3c2* is brain region‐dependent and is differentially influenced by traumatic experience, with the hippocampus exhibiting the strongest evidence for experience‐dependent modulation of corticosterone responsiveness.

#### 3.2.2 Relative Expression of *Nr3c1*

**FIGURE 3:**
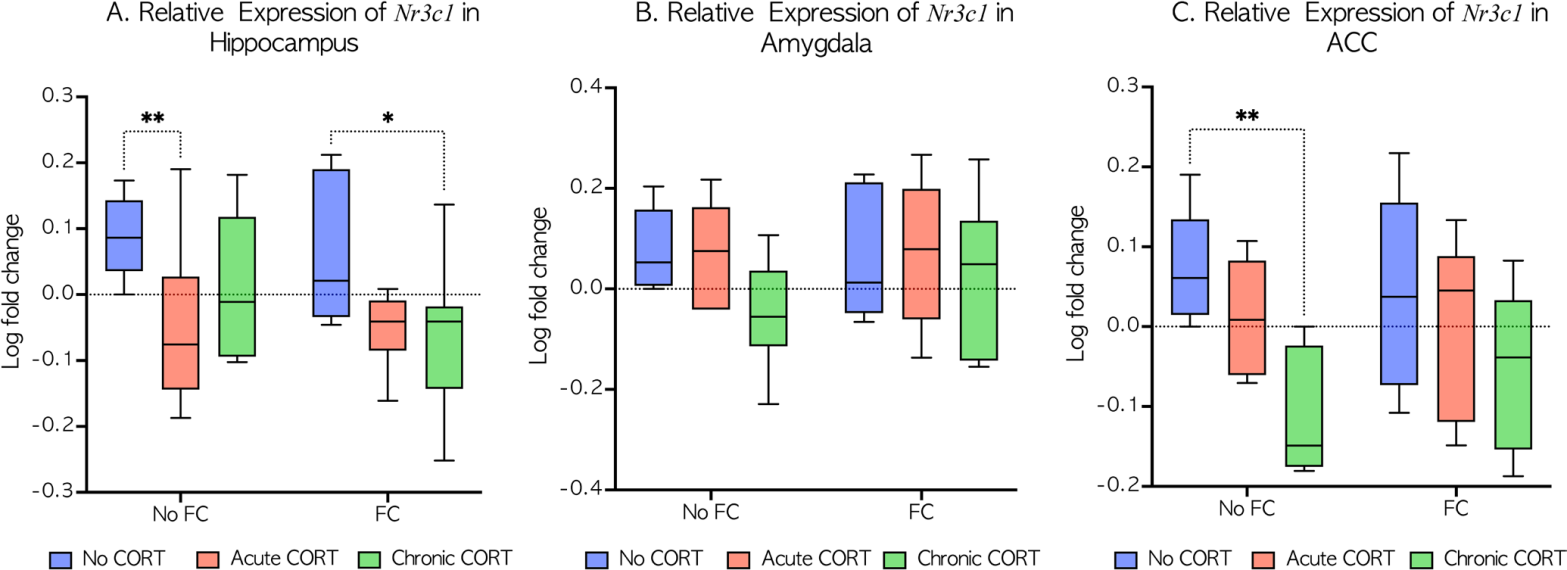
Effects of corticosterone treatment and FC on Nr3c1 mRNA expression in the hippocampus, amygdala, and ACC. Relative Nr3c1 mRNA expression was quantified by RT‐qPCR in the hippocampus (A), amygdala (B), and ACC (C) of mice receiving vehicle (No CORT), acute corticosterone (Acute CORT), or chronic corticosterone (Chronic CORT) treatment in the absence (No FC) or presence (FC) of FC. Gene expression was normalised to Hprt. Data are presented as box‐and‐whisker plots showing the median, interquartile range, and minimum‐to‐maximum values. Statistical analyses were performed separately for each brain region using ordinary two‐way ANOVA with Treatment (No CORT, Acute CORT, Chronic CORT) and Experience (No FC, FC) as fixed factors, followed by Tukey's multiple‐comparisons test. *n* = 9/group, except Chronic CORT (No FC) (*n* = 6) and Chronic CORT + FC for ACC (*n* = 8 due to tissue damage). Significant pairwise comparisons are indicated by brackets (*p < 0.05, **p < 0.01).

In the hippocampus, two‐way ANOVA revealed a significant main effect of Treatment (*F*(2,45) = 8.355, *p* = 0.0008), whereas neither the Treatment × Experience interaction (*F*(2,45) = 0.6196, *p* = 0.5427) nor the main effect of Experience (*F*(1,45) = 1.611, *p* = 0.2109) reached statistical significance (Figure 3A). These findings indicate that hippocampal *Nr3c1* expression was influenced primarily by corticosterone treatment, irrespective of FC. To further characterize the treatment effect, Tukey's multiple‐comparisons test was performed. Within the No FC condition, acute corticosterone treatment significantly reduced *Nr3c1* expression compared with vehicle‐treated mice (mean difference = 0.1402, 95% CI = 0.02978–0.2506, adjusted *p* = 0.0098). Within the FC condition, chronic corticosterone treatment also significantly reduced *Nr3c1* expression relative to vehicle‐treated animals (mean difference = 0.1237, 95% CI = 0.01332–0.2342, adjusted *p* = 0.0249). The comparison between vehicle‐ and acute‐treated mice following FC approached statistical significance (adjusted *p* = 0.0504). No significant differences were detected between acute and chronic corticosterone treatment within either Experience condition, and FC did not significantly alter *Nr3c1* expression within any treatment group.

In the amygdala, two‐way ANOVA revealed no significant Treatment × Experience interaction (*F*(2,45) = 0.6048, *p* = 0.5506), and neither the main effect of Treatment (*F*(2,45) = 2.557, *p* = 0.0888) nor Experience (*F*(1,45) = 0.2559, *p* = 0.6154) reached statistical significance (Figure 3B). Overall, these findings indicate that *Nr3c1* expression in the amygdala was not significantly influenced by corticosterone treatment or FC. Consistent with the overall ANOVA, Tukey's multiple‐comparisons analysis did not identify any significant pairwise differences among treatment groups within either the No FC or FC conditions. Likewise, FC did not significantly affect *Nr3c1* expression within the No CORT, Acute CORT, or Chronic CORT treatment groups.

In the ACC, two‐way ANOVA demonstrated a significant main effect of Treatment (*F*(2,44) = 8.495, *p* = 0.0008), whereas neither the Treatment × Experience interaction (*F*(2,44) = 1.114, *p* = 0.3374) nor the main effect of Experience (*F*(1,44) = 0.0131, *p* = 0.9093) was statistically significant (Figure 3C). These findings indicate that corticosterone treatment influenced ACC *Nr3c1* expression independently of FC. Post hoc Tukey's multiple‐comparisons analysis demonstrated that, within the No FC condition, chronic corticosterone‐treated mice exhibited significantly lower *Nr3c1* expression than vehicle‐treated animals (mean difference = 0.1888, 95% CI = 0.06796–0.3097, adjusted *p* = 0.0013). The comparison between acute and chronic corticosterone treatment approached statistical significance (adjusted *p* = 0.0518), whereas no other pairwise comparisons reached statistical significance. Within the FC condition, no significant differences among treatment groups were detected, and FC did not significantly alter *Nr3c1* expression within any treatment group.

Collectively, these findings demonstrate that *Nr3c1* regulation was primarily driven by corticosterone treatment rather than FC. Significant treatment effects were observed in both the hippocampus and ACC, whereas the amygdala exhibited no significant changes across experimental conditions. In the hippocampus, acute corticosterone significantly reduced *Nr3c1* expression under basal conditions, whereas chronic corticosterone significantly decreased *Nr3c1* expression following FC. Similarly, the ACC exhibited a treatment‐dependent reduction in *Nr3c1* expression that was most evident in chronically treated mice under basal conditions. In contrast, amygdalar *Nr3c1* expression remained stable regardless of corticosterone treatment or FC. Because no significant Treatment × Experience interactions were detected in any brain region, these findings indicate that glucocorticoid regulation of *Nr3c1* is predominantly treatment‐dependent rather than experience‐dependent, with the hippocampus and ACC exhibiting greater sensitivity to corticosterone exposure than the amygdala.

#### 3.2.3 Relative Expression of *Fkbp5*

**FIGURE 4:**
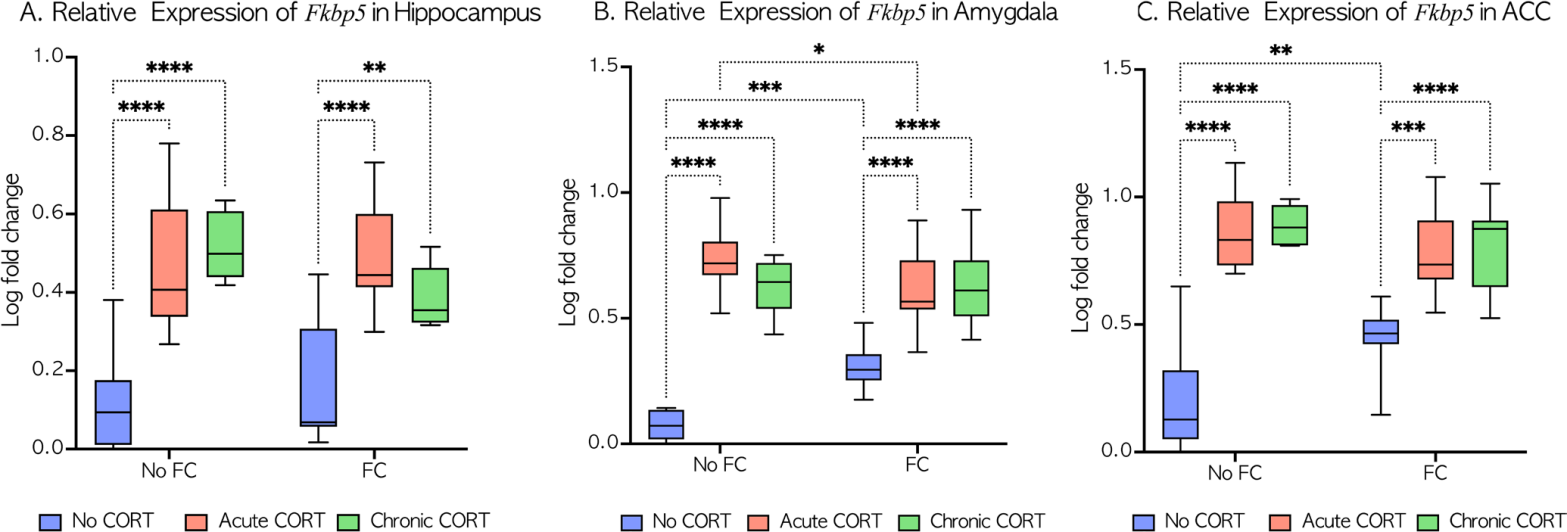
Effects of corticosterone treatment and FC on Fkbp5 mRNA expression in the hippocampus, amygdala, and ACC. Relative Fkbp5 mRNA expression was quantified by RT‐qPCR in the hippocampus (A), amygdala (B), and ACC (C) of mice receiving vehicle (No CORT), acute corticosterone (Acute CORT), or chronic corticosterone (Chronic CORT) treatment in the absence (No FC) or presence (FC) of FC. Gene expression was normalised to Hprt. Data are presented as box‐and‐whisker plots showing the median, interquartile range, and minimum‐to‐maximum values. Statistical analyses were performed separately for each brain region using ordinary two‐way ANOVA with Treatment (No CORT, Acute CORT, Chronic CORT) and Experience (No FC, FC) as fixed factors, followed by Tukey's multiple‐comparisons test *n* = 9/group, except Chronic CORT (No FC) (*n* = 6) and Chronic CORT + FC for ACC (*n* = 8 due to tissue damage). Significant pairwise comparisons are indicated by brackets (*p < 0.05, **p < 0.01, ***p < 0.001, ****p < 0.0001).

Two‐way ANOVA revealed a significant main effect of Treatment (*F*(2,45) = 33.10, *p* < 0.0001), whereas neither the Treatment × Experience interaction (*F*(2,45) = 1.886, *p* = 0.1635) nor the main effect of Experience (*F*(1,45) = 0.1913, *p* = 0.6639) reached statistical significance in the hippocampus (Figure 4A). These findings indicate that hippocampal *Fkbp5* expression was strongly influenced by corticosterone treatment, irrespective of FC. Tukey's multiple‐comparisons test demonstrated that, within the No FC condition, both acute and chronic corticosterone treatment significantly increased *Fkbp5* expression compared with vehicle‐treated mice (acute: mean difference = −0.3498, 95% CI = −0.5081 to −0.1915, adjusted *p* < 0.0001; chronic: mean difference = −0.4052, 95% CI = −0.5822 to −0.2282, adjusted *p* < 0.0001). Similarly, within the FC condition, both acute and chronic corticosterone treatment significantly increased *Fkbp5* expression relative to vehicle‐treated animals (acute: mean difference = −0.3332, 95% CI = −0.4915 to −0.1749, adjusted *p* < 0.0001; chronic: mean difference = −0.2298, 95% CI = −0.3881 to −0.0715, adjusted *p* = 0.0028). No significant differences were observed between acute and chronic corticosterone treatment under either Experience condition. Furthermore, FC did not significantly alter *Fkbp5* expression within any treatment group.

In the amygdala, two‐way ANOVA revealed a significant Treatment × Experience interaction (*F*(2,45) = 9.986, *p* = 0.0003), together with a highly significant main effect of Treatment (*F*(2,45) = 85.06, *p* < 0.0001), whereas the main effect of Experience was not significant (*F*(1,45) = 1.113, *p* = 0.2970) (Figure 4B). These findings indicate that the effect of corticosterone treatment on amygdalar Fkbp5 expression depended on FC. To further characterize this interaction, Tukey's multiple‐comparisons test was performed. Within the No FC condition, both acute and chronic corticosterone treatment significantly increased *Fkbp5* expression compared with vehicle‐treated mice (acute: mean difference = −0.6653, 95% CI = −0.8040 to −0.5265, adjusted *p* < 0.0001; chronic: mean difference = −0.5509, 95% CI = −0.7060 to −0.3957, adjusted *p* < 0.0001). Likewise, within the FC condition, both acute and chronic corticosterone treatment significantly increased *Fkbp5* expression relative to vehicle‐treated animals (acute: mean difference = −0.3077, 95% CI = −0.4465 to −0.1690, adjusted *p* < 0.0001; chronic: mean difference = −0.3199, 95% CI = −0.4586 to −0.1811, adjusted *p* < 0.0001). Acute and chronic corticosterone treatment did not differ significantly from each other under either Experience condition. Comparison of Experience levels demonstrated significantly higher *Fkbp5* expression in vehicle‐treated mice following FC than under basal conditions (mean difference = −0.2325, 95% CI = −0.3478 to −0.1172, adjusted *p* = 0.0002). In contrast, acute corticosterone‐treated mice exhibited significantly lower Fkbp5 expression following FC than under No FC conditions (mean difference = 0.1250, 95% CI = 0.0097–0.2404, adjusted *p* = 0.0342), whereas no significant effect of FC was detected within the chronic corticosterone group.

In the ACC, two‐way ANOVA demonstrated a significant Treatment × Experience interaction (*F*(2,44) = 6.253, *p* = 0.0041), together with a highly significant main effect of Treatment (*F*(2,44) = 59.18, *p* < 0.0001), whereas the main effect of Experience was not significant (*F*(1,44) = 0.3832, *p* = 0.5391) (Figure 4C). These findings indicate that the effect of corticosterone treatment on ACC Fkbp5 expression depended on FC. Post hoc Tukey's multiple‐comparisons analysis demonstrated that, within the No FC condition, both acute and chronic corticosterone treatment significantly increased *Fkbp5* expression relative to vehicle‐treated mice (acute: mean difference = −0.6749, 95% CI = −0.8584 to −0.4914, adjusted *p* < 0.0001; chronic: mean difference = −0.6909, 95% CI = −0.8960 to −0.4858, adjusted *p* < 0.0001). Similarly, within the FC condition, both acute and chronic corticosterone treatment significantly increased *Fkbp5* expression compared with vehicle‐treated animals (acute: mean difference = −0.3300, 95% CI = −0.5135 to −0.1465, adjusted *p* = 0.0002; chronic: mean difference = −0.3705, 95% CI = −0.5596 to −0.1814, adjusted *p* < 0.0001). Acute and chronic corticosterone treatment did not differ significantly from each other under either Experience condition. Comparison of Experience levels demonstrated significantly higher *Fkbp5* expression in vehicle‐treated mice following FC than under basal conditions (mean difference = −0.2502, 95% CI = −0.4026 to −0.0977, adjusted *p* = 0.0019), whereas FC did not significantly alter *Fkbp5* expression in either the acute corticosterone group (adjusted *p* = 0.2170) or the chronic corticosterone group (adjusted *p* = 0.4222).

Collectively, these findings demonstrate robust induction of *Fkbp5* by corticosterone across all three brain regions examined. The hippocampus exhibited a significant treatment‐dependent increase in *Fkbp5* expression that was independent of FC, indicating a consistent transcriptional response to corticosterone exposure regardless of behavioural experience. In contrast, both the amygdala and ACC displayed significant Treatment × Experience interactions, demonstrating that FC modified the magnitude of corticosterone‐induced *Fkbp5* expression. In both regions, acute and chronic corticosterone markedly increased *Fkbp5* expression relative to vehicle treatment under basal and FC conditions, whereas FC significantly increased *Fkbp5* expression only in vehicle‐treated animals. Additionally, in the amygdala, acute corticosterone‐treated mice exhibited lower *Fkbp5* expression following FC than under basal conditions, an effect not observed after chronic corticosterone treatment. Overall, these findings identify *Fkbp5* as the most robust glucocorticoid‐responsive transcript examined and demonstrate that its regulation is brain region‐dependent, with FC selectively modulating corticosterone‐induced transcriptional responses in the amygdala and ACC but not in the hippocampus.

## 4 Discussion

The present study investigated how acute and chronic corticosterone exposure influences trauma memory and the early transcriptional regulation of glucocorticoid signalling, including *Nr3c2*, *Nr3c1*, and *Fkbp5*, in brain regions critically involved in memory processing. Whereas acute corticosterone administration did not alter trauma memory formation, chronically treated mice failed to exhibit the significant increase in freezing and reduction in exploratory activity observed in vehicle‐treated and acutely treated animals. Although direct post‐FC comparisons between treatment groups were not significant, these findings suggest that prolonged, but not acute, glucocorticoid exposure attenuates trauma‐associated memory. This interpretation is consistent with previous evidence demonstrating that the effects of glucocorticoids on learning and memory critically depend on the duration and timing of hormone exposure [44, 45]. Moreover, comparable vocalisation responses during conditioning suggest that altered nociceptive sensitivity is unlikely to account for the observed behavioural phenotype.

To determine whether alterations in glucocorticoid *signalling* accompanied these behavioural differences, we examined the expression of *Nr3c2*, *Nr3c1*, and *Fkbp5* in the hippocampus, ACC, and amygdala. Among the genes examined, *Nr3c2* displayed the strongest dependence on the interaction between corticosterone exposure and FC, suggesting that MR‐associated transcription may represent the principal molecular response linking hormonal state with traumatic experience. In vehicle‐treated animals, hippocampal *Nr3c2* expression decreased following FC, consistent with models proposing that MR activation promotes the initial encoding of emotionally salient information and is subsequently followed by autoregulatory suppression of *Nr3c2* transcription, thereby facilitating closure of the early plasticity window [22, 23, 33]. Acute corticosterone modified this transcriptional response, whereas chronic corticosterone prevented the normal FC‐associated reduction in hippocampal *Nr3c2* expression. These findings suggest that prolonged glucocorticoid exposure alters MR‐dependent transcriptional regulation during the early stages of memory consolidation rather than simply enhancing glucocorticoid signalling. A distinct but complementary pattern emerged in the ACC. Unlike the hippocampus, chronic corticosterone treatment significantly increased *Nr3c2* expression following FC compared with chronically treated animals that did not undergo FC, an effect not observed after acute corticosterone exposure. Thus, repeated corticosterone exposure uniquely produced coordinated region‐specific regulation of *Nr3c2* across the hippocampus and ACC, preventing the physiological reduction of hippocampal expression while enhancing cortical expression following FC. Importantly, this coordinated molecular profile was exclusive to chronic corticosterone exposure, the only treatment associated with attenuated conditioning‐related behavioural responses. Although the present study cannot establish causality, these coordinated regional changes suggest that prolonged glucocorticoid exposure reorganises MR‐dependent regulation across hippocampal–prefrontal circuits involved in trauma‐memory processing rather than merely altering transcription within individual regions. In contrast, amygdalar *Nr3c2* expression remained comparatively stable. Neither Treatment nor Experience significantly influenced transcription; nevertheless, stable *Nr3c2* mRNA expression does not exclude functional amygdala adaptations, as chronic stress has been shown to increase amygdala excitability [46]. The isolated increase observed following acute corticosterone under basal conditions should be interpreted cautiously because the overall ANOVA was not significant. Together, these findings indicate that hippocampal and cortical MR transcription are substantially more responsive to prolonged glucocorticoid exposure than amygdalar MR transcription during the early post‐conditioning period.

**FIGURE 5:**
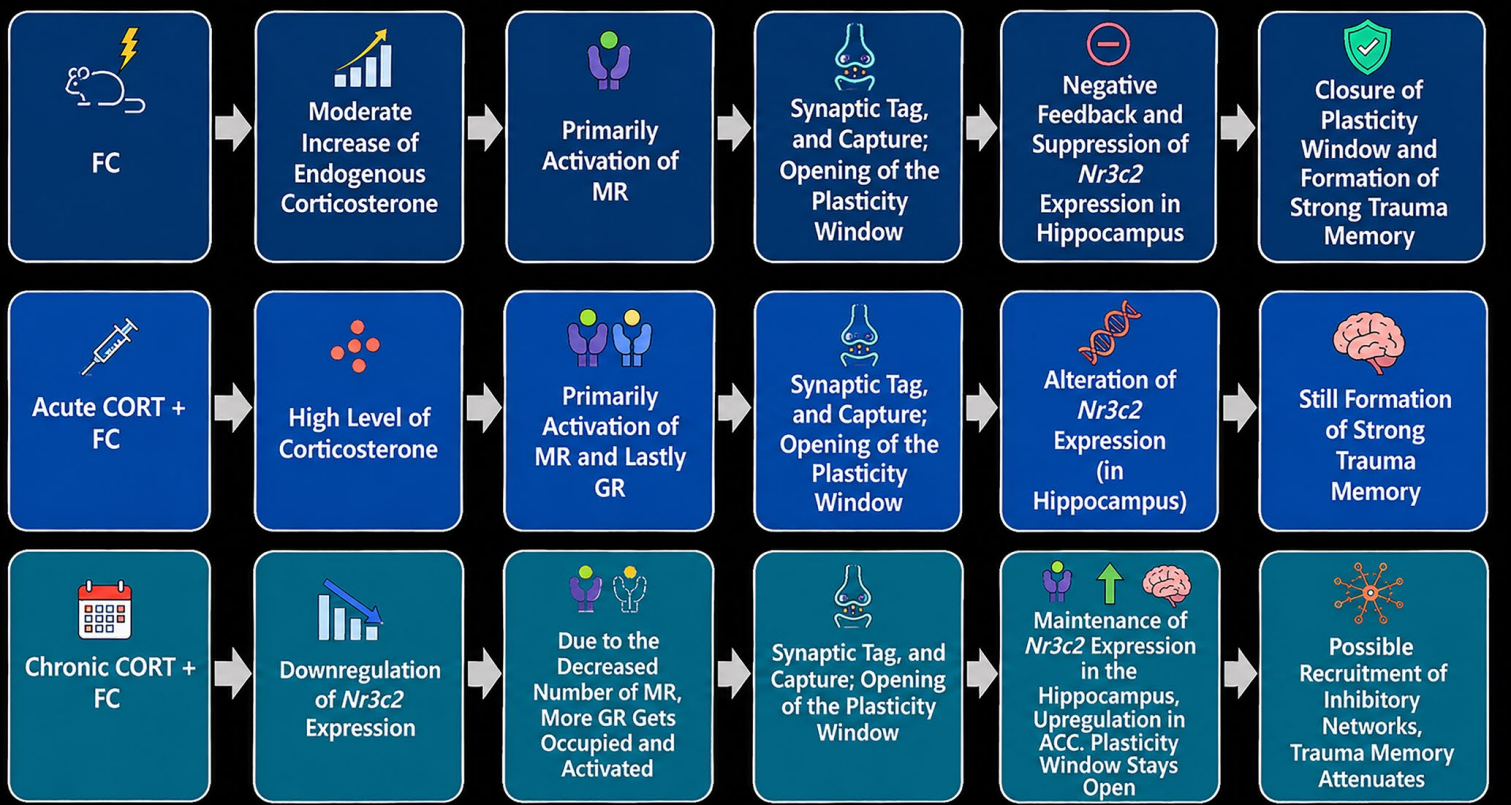
Proposed hypothesis illustrating a putative mechanism by which acute and chronic corticosterone exposure differentially influences trauma‐memory formation through regulation of glucocorticoid signalling. Under physiological conditions, FC induces a moderate increase in endogenous corticosterone. Because MRs exhibit approximately 6–10‐fold higher affinity for glucocorticoids than GRs, MRs are expected to be predominantly occupied and activated during the early phase of the stress response, whereas GR recruitment remains relatively limited [23, 27, 31]. Predominant MR activation promotes the opening of an early synaptic plasticity window and facilitates memory encoding [28, 33]. Based on the present findings, we propose that this physiological response is accompanied by autoregulatory suppression of hippocampal *Nr3c2* expression, contributing to closure of the plasticity window and the formation of a strong trauma memory. Acute corticosterone administration before FC transiently increases glucocorticoid availability and alters hippocampal *Nr3c2* expression without modifying the overall behavioural outcome, resulting in trauma‐memory formation comparable to vehicle‐treated animals. In contrast, repeated corticosterone exposure before FC is hypothesised to reduce MR availability through prior downregulation of *Nr3c2* expression. Consequently, during FC, a greater proportion of glucocorticoids may occupy and activate GRs, thereby shifting the MR/GR balance and preventing the normal FC‐associated suppression of hippocampal *Nr3c2* expression while promoting increased *Nr3c2* expression in the ACC. We further hypothesize that maintenance of hippocampal *Nr3c2* expression together with increased ACC *Nr3c2* expression prolongs MR‐dependent synaptic plasticity and facilitates recruitment of inhibitory cortical networks that attenuate excessive trauma‐memory formation. Thus, the coordinated region‐specific regulation of *Nr3c2* observed after chronic corticosterone exposure may represent an early molecular adaptation through which prolonged glucocorticoid exposure reshapes trauma‐memory processing. The proposed model integrates the present behavioural and transcriptional findings with the MR/GR balance hypothesis and should be regarded as a conceptual, testable framework rather than an experimentally demonstrated molecular mechanism [28, 33].

The coordinated region‐specific regulation of *Nr3c2* across the hippocampus and ACC forms the basis of the mechanistic model proposed in Figure 5. Under physiological conditions, FC induces transient MR activation in the hippocampus, promoting memory encoding followed by suppression of *Nr3c2* transcription, thereby facilitating timely closure of the plasticity window. Because MRs exhibit approximately 6–10‐fold higher affinity for corticosterone than GRs, they are expected to be predominantly occupied and activated during the early phase of the stress response, whereas GR recruitment remains comparatively limited [23, 27, 31]. We hypothesise that repeated corticosterone exposure before FC reduces MR availability through prior downregulation of *Nr3c2*, thereby shifting the MR/GR balance during subsequent trauma exposure towards greater GR activation. This altered receptor balance may prevent the normal autoregulatory suppression of hippocampal *Nr3c2* while simultaneously promoting increased cortical *Nr3c2* expression in the ACC. Consequently, maintenance of hippocampal *Nr3c2* expression together with increased ACC expression may prolong MR‐dependent synaptic plasticity and facilitate recruitment of inhibitory cortical networks that suppress excessive trauma‐memory formation. Although this mechanism remains speculative, the present findings identify a distinct region‐specific transcriptional signature associated specifically with prolonged glucocorticoid exposure and provide a biologically plausible framework for future studies investigating how chronic stress reshapes trauma‐memory circuitry. Because MR protein abundance, receptor occupancy, downstream signalling, and synaptic plasticity were not directly assessed, this model should be regarded as a testable hypothesis rather than a demonstrated molecular mechanism.

Unlike *Nr3c2*, regulation of *Nr3c1* was driven predominantly by corticosterone treatment rather than FC. Significant treatment effects were observed in the hippocampus and ACC, whereas amygdalar *Nr3c1* expression remained unchanged. These findings are consistent with the lower affinity of GRs for corticosterone, requiring higher ligand concentrations for activation than MRs [31], and support the interpretation that *Nr3c1* transcription primarily reflects pharmacological corticosterone exposure. Similarly, *Fkbp5* exhibited robust glucocorticoid responsiveness across all examined regions. In the hippocampus, both acute and chronic corticosterone increased *Fkbp5* expression irrespective of FC, whereas in the amygdala and ACC, *Fkbp5* regulation reflected the combined influence of corticosterone treatment and behavioural experience. Because acute and chronic corticosterone produced comparable *Fkbp5* induction despite distinct behavioural outcomes, changes in *Fkbp5* expression alone are unlikely to explain the attenuated behavioural phenotype. Instead, the behavioural effects of prolonged corticosterone exposure are more likely to reflect the coordinated regulation of multiple glucocorticoid‐*signalling* pathways rather than changes in any single downstream transcript.

Several limitations should be acknowledged. Behavioural differences were based primarily on within‐group comparisons, molecular analyses were limited to a single early time point, and receptor protein levels and circulating corticosterone were not assessed. Furthermore, only male mice were examined, and FC represents a simplified experimental model of trauma‐related memory. Future studies combining transcriptional, protein, endocrine, and circuit‐level analyses across multiple stages of memory consolidation will be required to determine whether the proposed mechanism directly contributes to the behavioural effects of prolonged glucocorticoid exposure.

In summary, chronic corticosterone exposure was associated with attenuated FC‐related behavioural responses and region‐specific regulation of glucocorticoid‐signalling genes during early memory consolidation. The principal molecular finding was the coordinated region‐specific regulation of *Nr3c2*, whereby chronic corticosterone prevented the normal FC‐associated reduction of hippocampal expression while simultaneously increasing *Nr3c2* expression in the ACC following FC. We propose that this coordinated MR‐dependent transcriptional profile represents an early molecular adaptation to prolonged glucocorticoid exposure that may modify communication within hippocampal–prefrontal circuits during trauma‐memory formation (Figure 5). Together, these findings identify a region‐specific *Nr3c2* transcriptional signature associated with chronic glucocorticoid exposure and provide a mechanistic framework for understanding how prolonged stress hormone elevation may reshape the molecular processes underlying traumatic memory formation.

## Funding

This work was supported by the Shota Rustaveli National Scientific Foundation of Georgia (FR/22‐26152).

## Conflicts of Interest

The authors declare no conflicts of interest.

## Data Availability

The datasets generated and/or analysed during this study are available from the corresponding author upon reasonable request.
